# Investigating nurses' acceptance of patients’ bring your own device implementation in a clinical setting: A pilot study

**DOI:** 10.1016/j.apjon.2023.100195

**Published:** 2023-02-05

**Authors:** Shuo-Chen Chien, Chun-You Chen, Chia-Hui Chien, Usman Iqbal, Hsuan-Chia Yang, Huei-Chia Hsueh, Shuen-Fu Weng, Wen-Shan Jian

**Affiliations:** a Graduate Institute of Biomedical Informatics, College of Medical Science and Technology, Taipei Medical University, Taipei, Taiwan; b Artificial Intelligence Research and Development Center, Wan Fang Hospital, Taipei Medical University, Taipei, Taiwan; c International Center for Health Information and Technology, College of Medical Science and Technology, Taipei Medical University, Taipei, Taiwan; d Department of Radiation Oncology, Wan Fang Hospital, Taipei Medical University, Taipei, Taiwan; e Office of Public Affairs, Taipei Medical University, Taipei, Taiwan; f Health ICT, Department of Health, Tasmania, Australia; g Global Health and Health Security Department, College of Public Health, Taipei Medical University, Taipei, Taiwan; h Department of Artificial Intelligence in Medicine, Professional Master Program, Taipei Medical University, Taipei, Taiwan; i Department of Pharmacy, Taipei Veterans General Hospital, Taipei, Taiwan; j Division of Endocrinology and Metabolism, Department of Internal Medicine, Taipei Medical University Hospital, Taipei, Taiwan; k Division of Endocrinology and Metabolism, Department of Internal Medicine, School of Medicine, College of Medicine, Taipei Medical University, Taipei, Taiwan; l School of Health Care Administration, Taipei Medical University, Taipei, Taiwan; m School of Gerontology Health Management, College of Nursing, Taipei Medical University, Taipei, Taiwan; n Research Center for Artificial Intelligence in Medicine, Taipei Medical University, Taipei, Taiwan; o Graduate Institute of Data Science, Taipei Medical University, Taipei, Taiwan

**Keywords:** Technology acceptance model, Bring your own device, Nurse acceptance, Internet of things, Smart hospital

## Abstract

**Objective:**

The popularity of the ​“bring your own device (BYOD)” ​concept has grown in recent years, and its application has extended to the healthcare field. This study was aimed at examining nurses’ acceptance of a BYOD-supported system after a 9-month implementation period.

**Methods:**

We used the technology acceptance model to develop and validate a structured questionnaire as a research tool. All nurses (*n* ​= ​18) responsible for the BYOD-supported wards during the study period were included in our study. A 5-point Likert scale was used to assess the degree of disagreement and agreement. Statistical analysis was performed in SPSS version 24.0.

**Results:**

The questionnaire was determined to be reliable and well constructed, on the basis of the item-level content validity index and Cronbach α values above 0.95 and 0.87, respectively. The mean constant values for all items were above 3.95, thus suggesting that nurses had a positive attitude toward the BYOD-supported system, driven by the characteristics of the tasks involved.

**Conclusions:**

We successfully developed a BYOD-supported system. Our study results suggested that nursing staff satisfaction with BYOD-supported systems could be effectively increased by providing practical functionalities and reducing clinical burden. Hospitals could benefit from the insights generated by this study when implementing similar systems.

## 1 Introduction

The use of health information technology, including electronic health records, computerized physician order entry systems, and bar code medication administration systems, has substantially improved healthcare quality by reducing repetitive tasks, preventing medication errors, and enhancing patient safety^1^, ^2^, ^3^, ^4^, ^5^. In recent years, the mobile health era has emerged and led to the integration of eHealth applications, driven by the rapid proliferation of mobile phones and personal electronic devices.^6^ The widespread availability of wireless infrastructure also enables the use of various current and emerging healthcare applications, such as bring your own device (BYOD).^7^^,^^8^

In the healthcare industry, BYOD allows professionals to use their personal devices for work purposes, including accessing patient records and performing job-associated tasks.^9^^,^^10^ In addition, BYOD allows patients to access their electronic health record data, communicate with their healthcare providers, schedule appointments, and even refill prescriptions through patient portals.^11^^,^^12^ The goal of BYOD is to improve the efficiency and quality of care by providing a more convenient and flexible option for both patients and providers.^13^ However, substantial security and privacy risks are associated with BYOD because personal devices may not be as secure as those provided by healthcare organizations.^14^ Additionally, users may be concerned about the perceived risk or uncertainty of using their own devices and the availability of services.^15^ Some studies have indicated that implementing BYOD could increase healthcare providers' burden.^16^^,^^17^ Although nurses using their own devices at work have been studied,^18^ the acceptance of providers, such as nursing staff toward patients using their own equipment during hospitalization, must also be explored.

The technology acceptance model (TAM) is a robust theoretical framework often used to explain why users accept or reject new technology.^19^ Many studies have widely used the TAM because of its ability to help understand user behavior.^20^ The TAM has also been widely applied in the healthcare industry to understand healthcare professionals' attitudes toward information systems.^21^ For example, Nguyen et al. have used the TAM to investigate the use of telehealth technologies in palliative care and have found that user acceptance is largely influenced by whether the new technology poses a substantial burden on providers and patients.^22^ Similarly, Syeda et al. have used an extended version of the TAM to study the acceptance of telemedicine services among rural populations in Pakistan.^23^ These studies have demonstrated that the TAM can be used to identify critical factors, such as perceived ease of use and perceived usefulness. Thus, to answer the study question and fill current gaps in existing research, we used TAM to examine the critical factors determining nurses' use of BYOD support systems.

We collaborated with a Taiwanese academic medical center in implementing a BYOD-supported system by retrofitting the original general wards. The BYOD-supported system was an Internet of Things-based system that allowed patients to control ward facilities by using their devices. After a 9-month implementation period, we used a TAM-based structured questionnaire to evaluate the attitudes among all nurses responsible for BYOD-supported wards. Finally, we determined the crucial factors influencing nurse acceptance of the BYOD-supported system. On the basis of our findings, we provided suggestions for hospital administrators designing similar systems.

## 2 Methods

### 2.1 Design of the BYOD-supported system

**Fig. 1:**
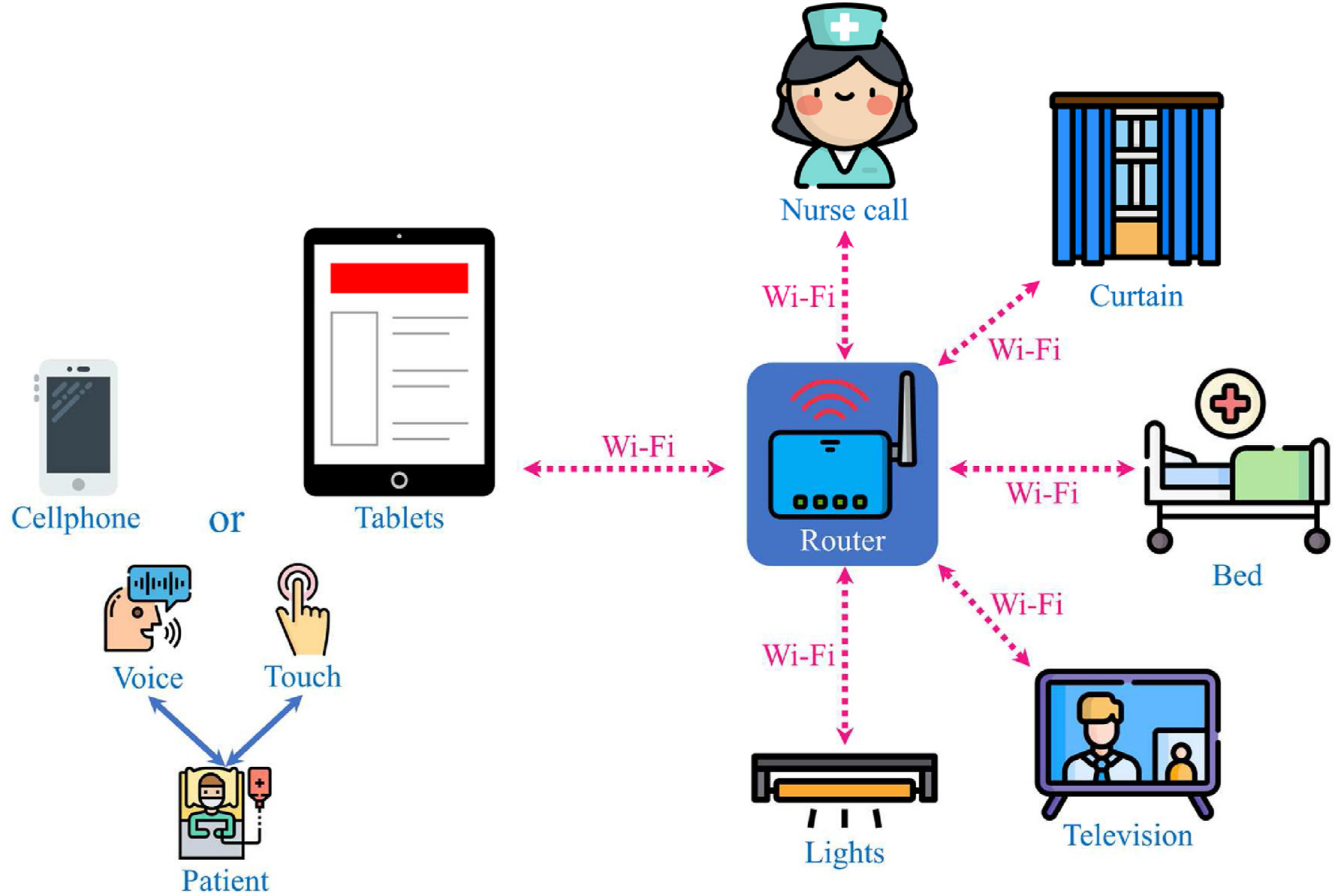
The BYOD-supported system architecture.

Traditionally, patients used switches and controllers to control ward facilities or required assistance from others. In the experimental setting of our smart hospital, when patients were hospitalized, they could download an app to use the BYOD-supported system, which allowed them to use voice commands or clicks to control equipment with their mobile devices remotely (Fig. 1). In this experimental study, two BYOD-supported wards were retrofitted from the general pediatric wards.

**Fig. 2:**
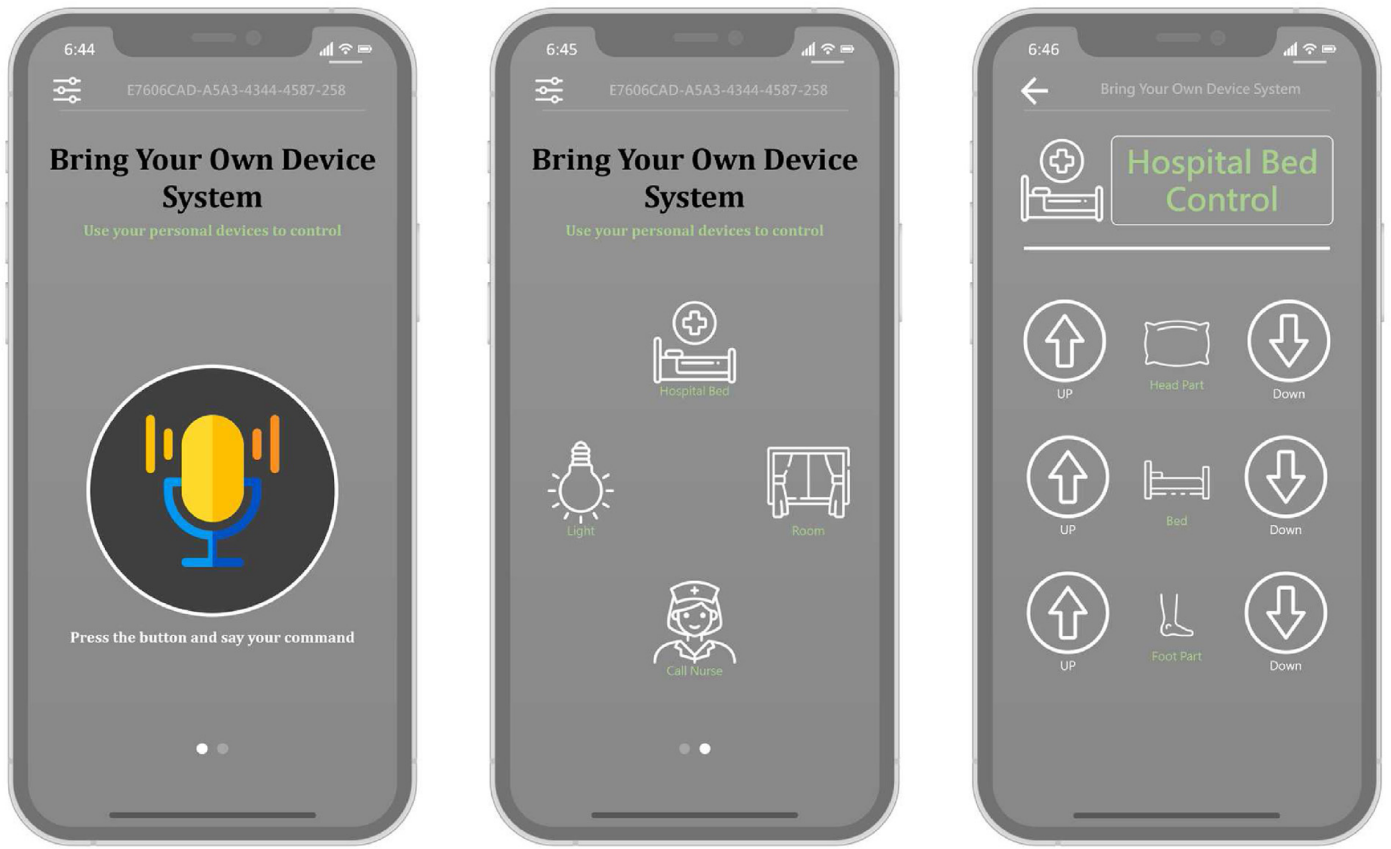
The bring your own device-supported system graphical user interface (GUI).

The IOS and Android system app for the BYOD-supported system was developed by using React Native, an open-source mobile application framework. Fig. 2 shows the BYOD-supported system's graphical user interface. Users could control the ward facilities in two ways: (1) speaking instructions while pushing the button or (2) using the icons. This app allowed patients to control ward facilities by using their mobile phones or tablets.

### 2.2 Technology acceptance model

The TAM is a set of theories developed by Fred Davis in 1989. TAM has been demonstrated to efficiently explain why people accept or reject a technology, particularly with respect to use behavior.^19^ Additionally, the TAM has been widely used to predict and interpret the acceptance of information technology systems under the assumption of rational behavior. In the TAM structure, perceived usefulness (the belief that using the technology will improve job performance) and perceived ease of use (the belief that using the technology is not difficult) are crucial factors that positively affect attitude toward using. Perceived usefulness is positively affected by perceived ease of use. Attitude toward using and perceived usefulness determine users' behavioral intentions and ultimately their actual system usage (Supplementary Fig. S1). We assessed the feasibility factors (construct) regarding the impact of a BYOD-supported system according to the original TAM through literature verification, including task characteristics (TC), perceived ease of use (PEOU), perceived usefulness (PU), attitude toward using (AT), user satisfaction (US), and behavioral intention to use (BI). Ten hypotheses (H0–H10) were tested to validate the research structure (Supplementary Fig. S2).

H1 *TC positively affects PEOU.*

H2 *TC positively affects PU.*

H3 *PEOU positively affects PU.*

H4 *PEOU positively affects AT.*

H5 *PU positively affects AT.*

H6 *PEOU positively affects US.*

H7 *PU positively affects US.*

H8 *PU positively affects BI.*

H9 *AT use positively affects BI.*

H10 *US positively affects BI.*

### 2.3 Questionnaire design and validation

We designed and used a structural questionnaire based on TAM theory (**Appendix file**). Five experts were invited to assess the content validity and reliability of the questionnaire before the formal implementation. For content validity, experts evaluated each question's importance, clarity, and correlation. The item-level content validity index (I-CVI), universal agreement among experts on the scale-level content validity index (S-CVI/UA), and the average of the scale-level content validity index (S-CVI/Ave)^24^ had values above 0.79, 0.8, and 0.9, thus indicating good content validity.^24^^,^^25^ In addition, the reliability was evaluated, and Cronbach α above 0.70 indicated consistent internal reliability.^26^ The questionnaire had two parts comprising basic information on the study objective and TAM constructs. The basic information included gender, age, degree, language, clinical grade, clinical experience, and daily usage frequency of the BYOD-supported system. The TAM constructs comprised TC, PEOU, PU, AT, US, and BI. The level of disagreement or agreement was assessed with a 5-point Likert scale (strongly agree, 5; agree, 4; neutral, 3; disagree, 2; and strongly disagree, 1).^27^

### 2.4 Data analysis

We used Pearson correlation (*r*) to analyze the correlations between research variables. An absolute value of *r* ​= ​below 0.39, 0.40–0.69, and 0.70–1.00 represented weak, moderate, and strong correlations, respectively.^28^

The relationships between dependent and independent variables in five models (M1–M5) were assessed with multiple regression analysis (Table 1).^29^ The variance inflation factor (VIF) calculated multicollinearity check was acceptable, with a value less than 10 ^30^. All statistical analyses were performed in the Statistical Package for the Social Sciences (SPSS version 24.0; IBM Corp, Armonk, New York).

**Table 1:** Models for multiple regression analysis.

Model	Variables		Hypothesis
	Dependent	Independent	
1	Perceived ease of use (PEOU)	Task characteristics (TC)	**H1**
2	Perceived usefulness (PU)	Task characteristics (TC)	**H2**
		Perceived ease of use (PEOU)	**H3**
3	Attitude toward using (AT)	Perceived ease of use (PEOU)	**H4**
		Perceived usefulness (PU)	**H5**
4	User satisfaction (US)	Perceived ease of use (PEOU)	**H6**
		Perceived usefulness (PU)	**H7**
5	Behavioral intention to use (BI)	Perceived usefulness (PU)	**H8**
		Attitude toward using (AT)	**H9**
		User satisfaction (US)	**H10**

### 2.5 Ethical considerations

This study was conducted according to the guidelines of the Declaration of Helsinki and approved by the Institutional Review Board of Taipei Medical University Research Ethics Board (IBR No. N201902057 and 20190508). Informed consent was obtained from all subjects.

## 3 Results

All nurses responsible for the two BYOD-supported wards during the study period were included as participants. Invalid questionnaires were excluded, thus leaving 18 valid questionnaires in our study.

### 3.1 Content validity and reliability results

The content validity assessment results, including I-CVI importance (0.99 ​> ​0.79), I-CVI clarity (0.98 ​> ​0.79), I-CVI correlation (0.95 ​> ​0.79), S-CVI/UA (0.91 ​> ​0.8), and S-CVI/Ave (0.97 ​> ​0.9), indicated good content validity. Meanwhile, Cronbach α among the six investigated constructs was above 0.7 (TC ​= ​0.93, PU ​= ​0.94, PEOU ​= ​0.87, AT ​= ​0.95, BI ​= ​0.94, US ​= ​0.97). The survey instrument was therefore considered reliable and well constructed. Furthermore, two constructs, attitude toward using and user satisfaction, had excellent internal consistency, with an α coefficient was greater than 0.95.

### 3.2 Respondent characteristics

We used frequency distributions to understand the basic personal characteristics of the participants, including sex, age, education, major language, clinical experience, and daily use frequency of the BYOD-supported system (Table 2). All respondents were female, held a bachelor's degree, and spoke Mandarin. In terms of age, nearly three-quarters (72.2%) were between 21 and 30 years of age, four (22.2%) were between 31 and 40 years of age, and one (5.6%) was 41 years or older.

**Table 2:** Respondents' demographic characteristics (*n* ​= ​18).

Characteristics	*n*	%
Gender		
Female	18	100.0
**Age (years)**		
21–30	13	72.2
31–40	4	22.2
41 or older	1	5.6
**Highest education level**		
Bachelor's degree	18	100.0
**Major language**		
Chinese	18	100.0
**Clinical grade**		
N1	8	44.4
N2	7	38.9
N3	3	16.7
**Clinical experience**		
0–5 years	9	50.0
6–10 years	5	27.8
11–15 years	2	11.1
16–20 years	1	5.6
21 years above	1	5.6
**The BYOD-supported system's daily usage frequency**		
Never used	2	11.1
1–5 times	15	83.3
6–10 times	1	5.6

Of the 18 respondents, nearly half (44.4%) were N1 grade and had more than five years of clinical experience. In terms of daily use of the bring your own device (BYOD)-supported system, most respondents (83.3%) used the system one to five times, whereas only one respondent (5.6%) used it six to ten times. However, two respondents (11.1%) never used the system during the study period.

### 3.3 Measurement models

Supplementary Table S1 shows the descriptive statistics of each construct variable. Among these six constructs, TC ranked highest, with a mean score of 4.11. Meanwhile, BI had the second highest-ranked score (4.04) among all constructs. PU ranked lowest, with a mean score of 3.78 overall.

Before the multiple linear regression analysis, we evaluated the relationships among the six constructs and observed strong positive correlations between BI and US (*r* ​= ​0.92), AT and BI (*r* ​= ​0.86), TC and PU (*r* ​= ​0.84), AT and US (*r* ​= ​0.82), PEOU and US (*r* ​= ​0.80), PU and AT (*r* ​= ​0.78), PEOU and AT (*r* ​= ​0.75), and PU and US (*r* ​= ​0.73), all at a significance level of *P* ​< ​0.01 (two-tailed).

### 3.4 Hypothesis testing results

We performed multiple regression analysis to explore the interactions between dependent and independent variables and determine the best prediction model (Supplementary Table S2). No collinearity problems (VIF < 10) were indicated by the auxiliary regression (M1: VIF ​= ​1.00; M2: VIF ​= ​1.43, 1.43; M3: VIF ​= ​1.52, 1.52; M4: VIF ​= ​1.52, 1.52; M5: VIF ​= ​2.70, 3.95, 3.29) among the models, which are described below.

In M1, TC and PEOU were the dependent and independent variables, respectively. TC effectively explained 26% of the total variation (adjusted *R*^2^ ​= ​0.26, *P* ​= ​0.02) and had a positive effect on PEOU (β ​= ​0.55, *t* ​= ​2.63; *P* ​= ​0.02), thus supporting **H1**.

In M2, TC and PEOU were independent variables, and PU was the dependent variable. Two independent variables (TC and PEOU) effectively explained 68% of the total variation (adjusted R^2^ ​= ​0.68, *P* ​< ​0.001). The TC positively affected PU (β ​= ​0.74, *t* ​= ​4.50; *P* ​< ​0.001), thus supporting **H2**. However, the correlation between PEOU and PU was insignificant (*P* ​= ​0.29 ​> ​0.01), thus not supporting **H3**.

In M3, PEOU and PU were independent variables, and AT was the dependent variable. Two independent variables, PEOU and PU, effectively explained 70% of the total variation (adjusted *R*^2^ ​= ​0.70, *P* ​< ​0.001). We observed positive effects of PEOU (β ​= ​0.45, *t* ​= ​2.81; *P* ​= ​0.013) and PU (β ​= ​0.52, *t* ​= ​3.19; *P* ​= ​0.006) on AT, thus supporting **H4** and **H5**.

In M4, the independent variables were the same as in M3, whereas the dependent variable (US) was different. A total of 70% of the total variation was effectively explained by two independent variables (adjusted *R*^2^ ​= ​0.70, *P* ​< ​0.001). Both PEOU (β ​= ​0.56, *t* ​= ​3.46; *P* ​= ​0.004) and PU (β ​= ​0.40, *t* ​= ​2.43; *P* ​= ​0.03) positively affected the US, thus supporting **H6** and **H7**.

**Fig. 3:**
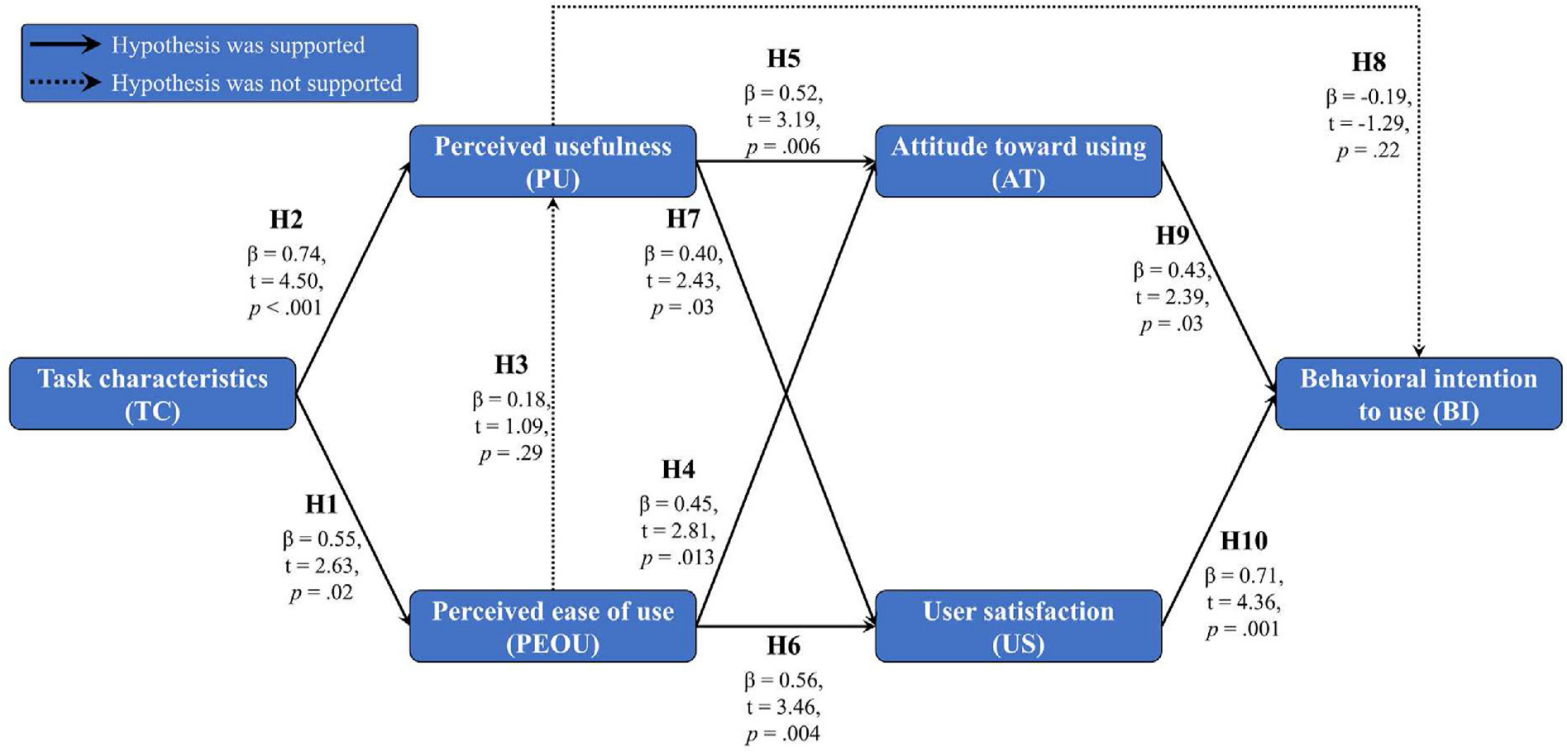
Hypothesis analysis results.

M5 included three independent variables: PU, AT, and US. The dependent variable was BI. PU, AT, and US effectively explained 86% of the total variation (adjusted *R*^2^ ​= ​0.86, *P* ​< ​0.001). Interestingly, in contrast to the finding that PU (*P* ​= ​0.22 ​> ​0.01) did not affect BI, **H9** and **H10** were supported because AT (β ​= ​0.43, *t* ​= ​2.39; *P* ​= ​0.03) and US (β ​= ​0.71, *t* ​= ​4.36; *P* ​= ​0.001) had positive effects on BI. Finally, on the basis of our results, we verified and summarized the ten research hypotheses in Fig. 3.

## 4 Discussion

In this study, we successfully investigated the acceptance of nursing staff toward patients' use of their own devices during hospitalization. To our knowledge, this is the first study that used the TAM to obtain deeper specific and contextualized insights for implementing BYOD services and evaluated nurses' attitudes after nine months of actual use. Previous researchers have examined the feasibility and patient acceptance of smart hospital wards^31^, ^32^, ^33^, ^34^, but nurses’ perspectives on BYOD services have not been determined. Our results showed that, on average, the six investigated constructs showed a high score of 3.95 out of 5, thus indicating that nurses positively accepted our BYOD system. The insights generated from our study should provide valuable information for hospitals considering implementing similar systems in the future.

The purpose of the TC assessment was to determine the effectiveness of the services provided by our BYOD system.^35^ TC received the highest score, with a mean of 4.11 and a range of 4.00–4.17, thereby indicating that, from the perspective of professional nurses, the BYOD system provided services required by patients during their hospital stay. These services allow patients, particularly those with disabilities, to conveniently control various elements of their hospital room, such as the bed, lights, and television, and to call for assistance from nurses,^36^ all of which are part of daily routines during hospitalization. Without these services, patients rely on their families or nurses to complete these tasks—a process that is inconvenient and inefficient.

In our study, PEOU and PU were significantly influenced by TC, thus suggesting that nurses tended to prioritize the functional capabilities of the system rather than its user-friendliness.^37^ Regarding the relationship between PEOU and PU, our previous study has indicated that an easy-to-use system improves patients' performance during hospitalization^12^; however, a similar effect on nurses' job performance was not observed in this research. Because the BYOD system was specifically designed for patients, nurses may understandably be more concerned about its stability. If the system is unstable or frequently malfunctions, nurses’ workloads could increase, thus potentially jeopardizing patient safety.

AT and US were positively influenced by PU and PEOU.^38^ Our auxiliary system that allowed patients to control facilities without the assistance of others reduced the burden on nurses, improved job performance, and elicited reasonable levels of satisfaction among both nurses and patients.^39^ Furthermore, AT and US significantly influenced BI. However, no significant relationship was found between BI and PU, in agreement with previous research.^40^ A potential explanation is that users may assess system features according to their needs before deciding to use the system. Nevertheless, users were required to spend some time before they could benefit from the system's usefulness; however, this aspect was difficult to measure.

In conclusion, our study provided opportunities to implement a BYOD-supported system in hospital wards. On the basis of our findings, we provided several recommendations for hospitals considering the design of similar systems. First, the system should be designed to enhance the daily work of nurses and physicians by providing practical functionality for routine tasks.^37^ Second, the system should aim to reduce the workload of nurses and increase the effectiveness of medical staff, without requiring them to spend additional time troubleshooting system failures.^39^^,^^41^ Third, ensuring that new users have a positive experience with the system can facilitate its use because initial ease of use is particularly important.^42^ By following these guidelines, hospitals can avoid medical staff burnout and improve patient safety.^43^

### 4.1 Limitations

This study had several limitations. One limitation was that the study objective considered only nurses responsible for the two BYOD-supported wards. Although the implementation lasted as long as nine months, the generalizability of the study findings may be limited by the small number of participants. Another limitation was that our research considered only the nurses’ perceptions. Future studies should also consider the viewpoints of physicians and IT staff. Finally, the current system included five functions that were not directly associated with the clinical workflow. Additional functions, such as medication reminders and fall notifications, will be added in the future.

## 5 Conclusions

This study successfully developed a BYOD-supported system that allowed patients to interact with ward facilities by using their personal devices in a Taiwanese academic medical center. After 9 months of implementation, a structured questionnaire based on the TAM was used to evaluate nurse acceptance of the BYOD-supported wards. Our results indicated that BYOD-supported systems should provide practical functionality and decrease the clinical burden to optimize nursing staff satisfaction effectively. These findings provided insight into the design of BYOD-supported systems as hospitals expand the traditional wards’ functionality.

## CRediT author statement

Conceptualization, Shuen-Fu Weng and Wen-Shan Jian; Data curation, Shuo-Chen Chien and Chun-You Chen; Formal analysis, Chia-Hui Chien; Funding acquisition, Chia-Hui Chien and Wen-Shan Jian; Investigation, Usman Iqbal; Methodology, Shuo-Chen Chien; Project administration, Usman Iqbal and Wen-Shan Jian; Resources, Shuo-Chen Chien, Chun-You Chen, and Wen-Shan Jian; Software, Chun-You Chen; Supervision, Shuen-Fu Weng and Wen-Shan Jian; Visualization, Usman Iqbal; Writing – original draft, Shuo-Chen Chien; Writing – review & editing, Shuo-Chen Chien and Shuen-Fu Weng. All authors had full access to all the data in the study, and the corresponding authors had final responsibility for the decision to submit for publication. The corresponding authors attest that all listed authors meet authorship criteria and that no others meeting the criteria have been omitted.

## Declaration of competing interest

The authors declare no conflict of interest.

## Funding

This study received no external funding.

## Ethics statement

This study was conducted according to the guidelines of the Declaration of Helsinki and approved by the Institutional Review Board of Taipei Medical University Research Ethics Board (IBR No. N201902057 and 20190508). Informed consent was obtained from all subjects.

## Data availability statement

The authors confirm that the data supporting the findings of this study are available within the article.
