# Corrigendum to “Investigating nurses' acceptance of patients' bring your own device implementation in a clinical setting: A pilot study” [Asia–Pacific J Oncol Nurs 10 (2023) 100195]

**DOI:** 10.1016/j.apjon.2024.100437

**Published:** 2024-05-20

**Authors:** Shuo-Chen Chien, Chun-You Chen, Chia-Hui Chien, Usman Iqbal, Hsuan-Chia Yang, Huei-Chia Hsueh, Shuen-Fu Weng, Wen-Shan Jian

**Affiliations:** a Graduate Institute of Biomedical Informatics, College of Medical Science and Technology, Taipei Medical University, Taipei, Taiwan; b Artificial Intelligence Research and Development Center, Wan Fang Hospital, Taipei Medical University, Taipei, Taiwan; c International Center for Health Information and Technology, College of Medical Science and Technology, Taipei Medical University, Taipei, Taiwan; d Department of Radiation Oncology, Wan Fang Hospital, Taipei Medical University, Taipei, Taiwan; e Office of Public Affairs, Taipei Medical University, Taipei, Taiwan; f Health ICT, Department of Health, Tasmania, Australia; g Global Health and Health Security Department, College of Public Health, Taipei Medical University, Taipei, Taiwan; h Department of Artificial Intelligence in Medicine, Professional Master Program, Taipei Medical University, Taipei, Taiwan; i Department of Pharmacy, Taipei Veterans General Hospital, Taipei, Taiwan; j Division of Endocrinology and Metabolism, Department of Internal Medicine, Taipei Medical University Hospital, Taipei, Taiwan; k Division of Endocrinology and Metabolism, Department of Internal Medicine, School of Medicine, College of Medicine, Taipei Medical University, Taipei, Taiwan; l School of Health Care Administration, Taipei Medical University, Taipei, Taiwan; m School of Gerontology Health Management, College of Nursing, Taipei Medical University, Taipei, Taiwan; n Research Center for Artificial Intelligence in Medicine, Taipei Medical University, Taipei, Taiwan; o Graduate Institute of Data Science, Taipei Medical University, Taipei, Taiwan

There are corrections required for the affiliations of the author, Usman Iqbal. The correct affiliation information for Usman Iqbal is as follows:

Author Name: Usman Iqbal

Affiliations:

^1^ School of Population Health, Faculty of Medicine and Health, University of New South Wales (UNSW), Sydney, Australia

^2^ Health ICT, Department of Health, Tasmania, Australia

^3^ Global Health & Health Security Department, College of Public Health, Taipei Medical University, Taiwan

We would like to apologise for any inconvenience caused.

