# Supplementary material for: Investigating nurses' acceptance of patients’ bring your own device implementation in a clinical setting: A pilot study
Source: Asia Pac J Oncol Nurs. 2023 Feb 5;10(3):100195. doi: 10.1016/j.apjon.2023.100195 (PMC10006526; doi:10.1016/j.apjon.2023.100195)
Supplement: Multimedia component 1 [file mmc1.docx]

Appendix/Supplementary

Supplementary Figures


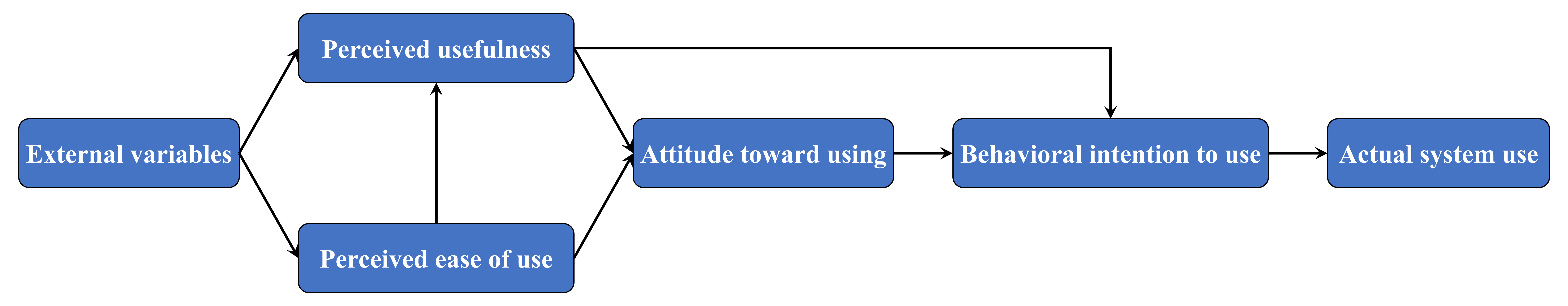


**Fig. S1.** The Technology Acceptance Model (TAM) architecture.


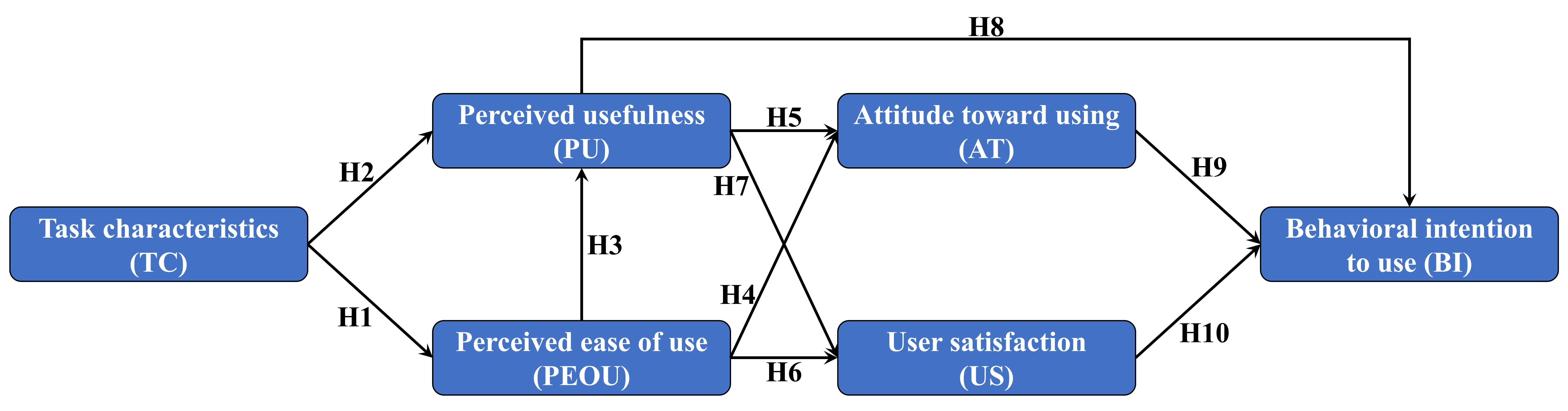


**Fig. S2.** The study hypothesis structure.

Supplementary Tables

**Table S1** Descriptive statistics among constructs.

| **Constructs** | **TC** | **PU** | **PEOU** | **AT** | **BI** | **US** |
| --- | --- | --- | --- | --- | --- | --- |
| **TC** | 1 |  |  |  |  |  |
| **PU** | 0.84^**^ | 1 |  |  |  |  |
| **PEOU** | 0.55^*^ | 0.58^*^ | 1 |  |  |  |
| **AT** | 0.64^**^ | 0.78^**^ | 0.75^**^ | 1 |  |  |
| **BI** | 0.47^*^ | 0.65^**^ | 0.65^**^ | 0.86^**^ | 1 |  |
| **US** | 0.63^**^ | 0.73^**^ | 0.80^**^ | 0.82^**^ | 0.92^**^ | 1 |
|  |  |  |  |  |  |  |
| **Number of questions (N)** | 3 | 6 | 5 | 5 | 4 | 3 |
| **Range** | 4.00 - 4.17 | 3.61 - 3.94 | 3.39 - 3.94 | 3.94 - 3.94 | 4.00 - 4.11 | 3.94 - 4.06 |
| **Mean** | 4.11 | 3.78 | 3.82 | 3.94 | 4.04 | 4.00 |
| Abbreviations: TC= Task characteristics; PEOU= Perceived ease of use; PU= Perceived usefulness; BI= Behavioral intention to use; US= User satisfaction; AT= Attitude toward using;  ^*^Correlation is at a significance level *p* < .05  ^**^Correlation is at a significance level *p* < .01 | | | | | | |

**Table S2** The multiple regression analysis results.

| **Model** | | **Unstandardized Coefficients** | | **Standardized Coefficients** | **t** | **Sig.**  ***P* values** | **VIF** |
| --- | --- | --- | --- | --- | --- | --- | --- |
|  |  | **B** | **Std. Error** | **Beta** |  |  |  |
| **1** | (Constant) | 10.46 | 3.33 |  | 3.14 | < .01 |  |
|  | Task characteristics (H1) | 0.70^*^ | 0.27 | 0.55 | 2.63 | .02 | 1.00 |
|  | Dependent variable: *PEOU* (R² = 0.30; Adjusted R² = 0.26; F = 6.94, *p* = .02) | | | | | | |
| **2** | (Constant) | -0.32 | 4.24 |  | -0.8 | .94 |  |
|  | TC (H2) | 1.44^***^ | 0.32 | 0.74 | 4.50 | < .001 | 1.43 |
|  | PEOU (H3) | 0.27 | 0.25 | 0.18 | 1.09 | .29 | 1.43 |
|  | Dependent variable: *PU* (R² = 0.72; Adjusted R² = 0.68; F = 6.49, *p* < .001) | | | | | | |
| **3** | (Constant) | 1.83 | 2.85 |  | 0.64 | .53 |  |
|  | PEOU (H4) | 0.50^*^ | 0.18 | 0.45 | 2.81 | .013 | 1.52 |
|  | PU (H5) | 0.38^**^ | 0.12 | 0.52 | 3.19 | .006 | 1.52 |
|  | Dependent variable: *AT* (R² = 0.74; Adjusted R² = 0.70; F = 21.63, *p* < .001) | | | | | | |
| **4** | (Constant) | -0.34 | 1.94 |  | -0.18 | .86 |  |
|  | PEOU (H6) | 0.42^**^ | 0.12 | 0.56 | 3.46 | .004 | 1.52 |
|  | PU (H7) | 0.19^*^ | 0.08 | 0.40 | 2.43 | .03 | 1.52 |
|  | Dependent variable: *US* (R² = 0.74; Adjusted R² = 0.70; F = 21.02, *p* < .001) | | | | | | |
| **5** | (Constant) | 2.28 | 1.40 |  | 1.63 | .126 |  |
|  | PU (H8) | -0.11 | 0.08 | -0.19 | -1.29 | .22 | 2.70 |
|  | AT (H9) | 0.33^*^ | 0.14 | 0.43 | 2.39 | .03 | 3.95 |
|  | US (H10) | 0.81^**^ | 0.19 | 0.71 | 4.36 | .001 | 3.29 |
|  | Dependent variable: BI (R² = 0.89; Adjusted R² = 0.86; F = 37.13, *p* < .001) | | | | | | |

Abbreviations: TC= Task characteristics; PEOU= Perceived ease of use; PU= Perceived usefulness; BI= Behavioral intention to use; US= User satisfaction; AT= Attitude toward using; VIF = Variance inflation factor;
^*^significant at *p* < .05; ^**^significant at *p* < .01; ^***^significant at *p* < .001.

Research questionnaire

Dear Sir/Madam:

This academic questionnaire aimed to evaluate your acceptance of the BYOD-supported system in the ward after actual use. Please answer the following questions according to your own feelings.

**The “BYOD-supported system“ allows patients to operate ward facilities (e.g., beds, lights, TVs, curtains) using a single device without assistance. Our study aims to use the technology acceptance model (TAM) to determine the factors why people accept or reject the BYOD-supported system.**

The results of this study are for academic use only. The information which you provide will be absolutely confidential. Please rest assured that to answer the questions. Your kind support and cooperation will be critical for successfully completing this research. Thank you very much for taking the time to finish the questionnaire.

Best regards!

**【BYOD-supported system】**

The BYOD-supported system allows patients to control the ward facilities (e.g., beds, lights, TVs, curtains) with their own devices.

**【Construct I. Task characteristic】**

The nurse subjectively considers that specific task characteristics of the BYOD-supported system were appropriate during the patient's hospitalization.

**【Construct II. Perceived usefulness】**

The nurse believes that the implementation of a BYOD-supported system can provide essential assistance and increase job performance.

**【Construct III. Perceived ease of use】**

The nurse believes the BYOD-supported system will not be difficult to learn and use.

**【Construct IV.** **Behavioral intention to us**e**】**

The personal favor of the BYOD-supported system for nurses, who have positive or negative comments on the system.

**【Construct V. Attitude toward using】**

Nurses will consistently use the BYOD-supported system in the future under their self-consciousness.

**【Construct VI. User satisfaction】**

The nurse satisfaction with the system after implementing a BYOD-supported system.

**【Part One】Questionnaire Content**

The following multiple-choice questions with a single answer have “Strongly Agree”, “Agree”, “Neutral“, “Disagree”, and “Strongly Disagree” five options, respectively. Please choose the answer identical to your own feelings according to the description of the question.

| **Construct I. Task characteristic** | | | | | |
| --- | --- | --- | --- | --- | --- |
|  | Strongly Agree | Agree | Neutral | Disagree | Strongly Disagree |
| A1. I think the BYOD-supported system could make me easier to operate the ward facilities. |  |  |  |  |  |
| A2. I think the BYOD-supported system can operate the ward facilities rapidly. |  |  |  |  |  |
| A3. Overall, I think the function provided by the BYOD-supported system is suitable for my requirements. |  |  |  |  |  |

| **Construct II. Perceived usefulness** | | | | | |
| --- | --- | --- | --- | --- | --- |
|  | Strongly Agree | Agree | Neutral | Disagree | Strongly Disagree |
| B1. I think it is pretty helpful for me while using the BYOD-supported system. |  |  |  |  |  |
| B2. I think the BYOD-supported system can improve work efficiency. |  |  |  |  |  |
| B3. I think the BYOD-supported system can shorten the treatment period. |  |  |  |  |  |
| B4. I think the BYOD-supported system is helpful for my routine task. |  |  |  |  |  |
| B5. I think the BYOD-supported system can simplify the operation of the ward facilities. |  |  |  |  |  |
| B6. Overall, I think the practicability of the BYOD-supported system is relatively high. |  |  |  |  |  |

| **Construct III. Perceived ease of use** | | | | | |
| --- | --- | --- | --- | --- | --- |
|  | Strongly Agree | Agree | Neutral | Disagree | Strongly Disagree |
| C1. I think using the BYOD-supported system to operate the ward facilities is easy. |  |  |  |  |  |
| C2. I think learning how to use the BYOD-supported system is simple. |  |  |  |  |  |
| C3. I need to spend more time than expected learning how to operate the BYOD-supported system properly. |  |  |  |  |  |
| C4. I think the BYOD-supported system is straightforward to use. |  |  |  |  |  |
| C5. Overall, Using the BYOD-supported system is easy. |  |  |  |  |  |

| **Construct IV. Behavioral intention to use** | | | | | |
| --- | --- | --- | --- | --- | --- |
|  | Strongly Agree | Agree | Neutral | Disagree | Strongly Disagree |
| D1. I am happy to use the BYOD-supported system. |  |  |  |  |  |
| D2. I am willing to use the BYOD-supported system. |  |  |  |  |  |
| D3. I think it is appropriate to implement the BYOD-supported system in hospital wards. |  |  |  |  |  |
| D4. I think the BYOD-supported system is not applicable. |  |  |  |  |  |
| D5. Overall, I think the BYOD-supported system's advantages outweigh the disadvantages. |  |  |  |  |  |

| **Construct V. Attitude toward using** | | | | | |
| --- | --- | --- | --- | --- | --- |
|  | Strongly Agree | Agree | Neutral | Disagree | Strongly Disagree |
| E1. I think it is worth using the BYOD-supported system. |  |  |  |  |  |
| E2. I am willing to spend more time learning how to use the BYOD-supported system since it is helpful to me. |  |  |  |  |  |
| E3. I would recommend those other people to use the BYOD-supported system. |  |  |  |  |  |
| E4. I am willing to use the BYOD-supported system in the future. |  |  |  |  |  |

| **Construct VI. User satisfaction** | | | | | |
| --- | --- | --- | --- | --- | --- |
|  | Strongly Agree | Agree | Neutral | Disagree | Strongly Disagree |
| F1. I am satisfied with the way using the BYOD-supported system. |  |  |  |  |  |
| F2. I am satisfied with the function provided by the BYOD-supported system. |  |  |  |  |  |
| F3. Overall, I am satisfied with the BYOD-supported system. |  |  |  |  |  |

**【Part Two】Basic information**

- 1. Sex:

□Male; □Female

- 1. Your age:

□ 20–40-years-old; □ 40–60-years-old; □ 60-years-old or older

- 1. The highest degree you have completed:

□ Senior high school; □ Bachelor's degree; □ Master's degree or above

- 1. Major language:

□ Chinese; □ English; □ Other

- 1. Clinical Grade:

□ N1; □ N2; □ N3; □ N4; □ Other

- 1. The daily use frequency of the voice smart care system:

□ Never used; □ 1~5 times; □ 6~10 times; □ 11~15 times; □ 16~20 times
